# Intentional and automatic numerical processing as predictors of mathematical abilities in primary school children

**DOI:** 10.3389/fpsyg.2015.00375

**Published:** 2015-03-31

**Authors:** Violeta Pina, Alejandro Castillo, Roi Cohen Kadosh, Luis J. Fuentes

**Affiliations:** ^1^Departamento de Psicología Básica y Metodología, Universidad de Murcia, MurciaSpain; ^2^Department of Experimental Psychology, University of Oxford, OxfordUK

**Keywords:** mathematical abilities, size comparison task, numerical comparison task, congruency effect, numerical distance effect, inhibitory control, primary school children

## Abstract

Previous studies have suggested that numerical processing relates to mathematical performance, but it seems that such relationship is more evident for intentional than for automatic numerical processing. In the present study we assessed the relationship between the two types of numerical processing and specific mathematical abilities in a sample of 109 children in grades 1–6. Participants were tested in an ample range of mathematical tests and also performed both a numerical and a size comparison task. The results showed that numerical processing related to mathematical performance only when inhibitory control was involved in the comparison tasks. Concretely, we found that intentional numerical processing, as indexed by the numerical distance effect in the numerical comparison task, was related to mathematical reasoning skills only when the task-irrelevant dimension (the physical size) was incongruent; whereas automatic numerical processing, indexed by the congruency effect in the size comparison task, was related to mathematical calculation skills only when digits were separated by small distance. The observed double dissociation highlights the relevance of both intentional and automatic numerical processing in mathematical skills, but when inhibitory control is also involved.

## Introduction

In recent years, there has been an increasing interest on the cognitive and neural mechanisms that underlay children’s mathematical performance. Of particular relevance is to determine whether individual differences in the processing of numerical information can explain how well elementary school children perform mathematical tasks that require different abilities, and whether such differences in numerical processing may explain specific learning disorders in mathematics (De Smedt et al., 2010; Butterworth et al., 2011). This issue is important not only from a theoretical point of view, but also for the design and assessment of new educational interventions that promote numerical processing (see De Smedt et al., 2013, for a review). To accomplish that aim we first need a task (or a set of tasks) that allows researchers to assess how children process numerical information, and how such processing develops with age and/or schooling years. Then, we need to assess children’s performance in a rather ample range of mathematical tests that are supposed to tap different mathematical abilities, from simple arithmetic operations to more complex word arithmetic problems. Here, we assessed numerical processing through numerical Stroop tasks (Henik and Tzelgov, 1982; Tzelgov et al., 1992; Girelli et al., 2000; Rubinsten et al., 2002; Schwarz and Ischebeck, 2003; Cohen Kadosh et al., 2011). Also, to assess different mathematical areas we used the Spanish version of the Woodcock Johnson III Tests of Achievement battery (Muñoz-Sandoval et al., 2005), which was primarily validated for use with participants from 6 to 13 years in Spain (see Diamantopoulou et al., 2012).

### The Numerical Stroop Task

Numerals convey rich information regarding semantic and physical attributes. In order to determine how that information is processed and what factors modulate such processing, authors have designed tasks in which participants are required to respond to certain dimensions of the number stimulus, such as the numerical magnitude and/or the physical size. These tasks allow researchers to assess how those dimensions are perceived and how they interact with each other (Schwarz and Ischebeck, 2003). By combining the physical size and the numerical value dimensions, and making one dimension task-relevant and the other task-irrelevant (like in Stroop-like paradigms), it may be determined how each dimension interferes with the intentional processing of the other. Moreover, Stroop-like paradigms have been probed useful to assess whether such processing can occur unintentionally in an automatic way (Tzelgov and Ganor-Stern, 2005).

Several types of numerical Stroop tasks have been amply used for that purpose (e.g., Henik and Tzelgov, 1982; Tzelgov et al., 1992; Girelli et al., 2000; Rubinsten et al., 2002; Rubinsten and Henik, 2005; Tang et al., 2006; Cohen Kadosh et al., 2007; Holloway and Ansari, 2009; Heine et al., 2010; Bugden and Ansari, 2011; Wang et al., 2013). In a typical experiment, two digits are displayed on the center of the screen. Participants are asked to compare both digits and indicate which one is bigger in size (hereafter the *size comparison task*) or in numerical magnitude (hereafter the *numerical comparison task*). In the size comparison task, the digit size is the relevant dimension and the numerical value the to-be-ignored dimension. In the numerical comparison task the numerical value is the relevant dimension and the physical size the to-be-ignored dimension. As both dimensions are independently manipulated, two crucial experimental conditions can be created for each task. In the congruent condition the bigger digit has also the greater numerical magnitude (e.g., 3 6). In the incongruent condition, the bigger digit has the smaller numerical magnitude and vice versa (e.g., 3 6). Congruency effects in both tasks can be computed by subtracting performance (i.e., reaction times, RTs; errors) in the congruent condition from the incongruent condition. Positive values reflect that the to-be-ignored dimension of the stimulus has been processed automatically. The fact that both the to-be-ignored numerical value (Besner and Coltheart, 1979) and the to-be-ignored physical size (Henik and Tzelgov, 1982) produce interference in the responses to the physical size and the numerical magnitude comparisons, respectively, suggests that continuous quantities share a common system. In addition, the congruency effect can reveal how efficient is the attentional control system both to detect conflict and to inhibit interference from the to-be-ignored dimension. An efficient system is one that resolves conflict caused by the irrelevant dimension without a high proportion of errors or excessive long RTs in responses to incongruent trials.

The pair of digits may also differ according to the numerical distance. For instance, the two digits can be separated by distance 1 (e.g., 3 vs. 4), distance 2 (e.g., 3 vs. 5), distance 3 (e.g., 3 vs. 6), and so on. It has been assumed that the smaller the numerical distance between the two digits, the longer the RTs (Moyer and Landauer, 1967). This effect has been interpreted by assuming that people represent numbers in an ordered mental number line (Dehaene and Cohen, 1995). As the digits are closer in the mental line, their representations might overlap and therefore the difficulty for number comparison increases.

Several relevant indexes can be computed when both numerical and size comparison tasks are performed in a single experiment. The first index concerns the *congruency effect* in the numerical comparison task, which refers to the interference caused by the task-irrelevant physical size of the stimuli. The need to ignore the task-irrelevant dimension would involve the attentional control mechanisms. Thus, a rather small numerical congruency effect is expected if the attentional control mechanisms are very efficient to deal with task-irrelevant information. Nonetheless, a lack of interference or a small effect may be due to reduced automaticity of the to-be-ignored dimension. This might stem from either an immature or damaged cognitive system. An inspection to the error rate can be of much help in accounting for such extremely small interference effects. The second index concerns the *numerical Stroop effect* in the size comparison task, which has been long thought of as a marker of *automatic numerical processing* (Henik and Tzelgov, 1982; Tzelgov et al., 1992; Rubinsten et al., 2002; Szűcs et al., 2007; Cohen Kadosh et al., 2008a,b, 2011, 2012; Rubinsten and Henik, 2009; Heine et al., 2010; Santens and Verguts, 2011). The better the numerical abilities are, the larger the numerical Stroop effect is expected, which indicates greater level of automaticity in numerical processing (Girelli et al., 2000; Rubinsten et al., 2002; Rubinsten and Henik, 2005; Cohen Kadosh et al., 2007). The third index refers to the *numerical distance effect*, which is usually interpreted as a marker of *intentional numerical processing* (Rubinsten et al., 2002). The better the numerical abilities are, the smaller the numerical distance effect (Rubinsten et al., 2002; Holloway and Ansari, 2009; Heine et al., 2010; Mussolin et al., 2010; Bugden and Ansari, 2011).

The task-irrelevant information in both the size and the numerical comparison tasks may cause inhibitory processes to modulate the intentional and/or the automatic markers of numerical processing. Thus, the distance effect in the numerical comparison task can be affected by whether the task-irrelevant dimension of the stimulus is incongruent (requiring attention-dependent inhibitory control) or congruent (inhibitory control is not required). Similarly, the numerical Stroop effect in the size comparison task can be affected by whether the numerical values activate competing (overlapping) numerical representations (small numerical distance), involving inhibitory control, or such competence is minimal as it happens with larger numerical distances. Importantly for the present study is to determine whether mathematical performance is related to numerical processing *per se*, and/or to the efficiency of inhibitory control mechanisms (see Soltész et al., 2011; Gilmore et al., 2013).

In the following sections we will address how the two types of numerical processing develop with age and how they relate to mathematical abilities in primary school children.

### Development of Numerical Processing

A main goal of the present research is to investigate how intentional and automatic numerical processing relates to mathematical performance in children that were in grades 1–6. Several studies have shown that intentional numerical processing is present in an ample range of ages, ranging from kindergarten (e.g., Sasanguie et al., 2012), primary school (Girelli et al., 2000; Rubinsten et al., 2002; Sasanguie et al., 2012) to adult age (see Noël et al., 2005, for a review). However, automatic numerical processing, as indexed by numerical Stroop effect in the size comparison task, seems to emerge later on. In the Western culture, children of first grade show numerical Stroop effects only if they are tested at the end of first grade (Rubinsten et al., 2002; Bugden and Ansari, 2011), but not if they are tested at the beginning of the first grade (Girelli et al., 2000; Rubinsten et al., 2002). Children may have acquired enough experience during the first schooling year so that by the end of the academic year numerical processing has become automatic. These results suggest that automatization of numerical processing develops with age.

### Numerical Processing and Math Abilities

Individual differences in basic numerical processing of children of an ample range of age correlate with individual differences in some mathematical abilities. This contention is supported by the use of an ample range of tasks that measure numerical processing, different tests that measure mathematical abilities, and children with and without a diagnosis of dyscalculia (Halberda et al., 2008; De Smedt et al., 2009; Holloway and Ansari, 2009; Heine et al., 2010; Bugden and Ansari, 2011; Sasanguie et al., 2012; Göbel et al., 2014; van Marle et al., 2014). For instance, De Smedt et al. (2009) used a numerical comparison task but did not manipulate any other dimension of the stimuli (e.g., the physical size), and therefore only intentional numerical processing could be assessed. They also tested mathematics achievement in a sample of first year children, and then when children were in the second year. Their results showed that the numerical distance effect predicted children’s mathematics achievement in the second year. The relationship between intentional numerical processing and math abilities was extended to children in kindergarten, first, second, and sixth year, although the effect was larger in the younger than in the older children (Sasanguie et al., 2012). In a more recent study, Sasanguie et al. (2013; see also Vanbinst et al., 2012) extended such relationship to a timed math test that required simple additions, subtractions, multiplications, and divisions. The results confirmed the previous relationship between general mathematical performance and intentional processing. Better performance in the numerical comparison task was predictive of higher score on the timed math test 1 year later. The use of different mathematical tests raises the possibility that not all mathematical abilities are similarly related to intentional numerical processing. For instance, Holloway and Ansari (2009) observed that children aged 6–8 years that scored higher in some mathematical tests showed also smaller numerical distance effects. Importantly, the numerical distance effect correlated more strongly with scores in the mathematical fluency test than with scores in calculation or the composite mathematical measure (see also Bugden and Ansari, 2011). These results suggest that intentional numerical processing is not a good predictor of performance in all the mathematical abilities explored.

Why does smaller distance effects lead to better performance in mathematics? Vanbinst et al. (2012) suggest that smaller distance effects are associated with more precise mapping between Arabic numerical representations and their magnitudes. This is beneficial to mathematical performance because it reflects better understanding of the relationship between numbers and the representation of quantity, otherwise arithmetic would be a mere memory retrieval exercise (Griffin, 2002; Robinson et al., 2002).

Some other studies have used comparison tasks that manipulated both numerical magnitude and physical size of numbers (Heine et al., 2010; Bugden and Ansari, 2011). Bugden and Ansari (2011) tested children in first and second grade in two mathematical tests, fluency, and calculation. However, the congruency effect in the size comparison task (i.e., automatic numerical processing) did not correlate with any of the mathematical tests. Heine et al. (2010) classified children in second and third grade as high, normal, and low mathematical achievement groups according to scores in a general mathematical test. The results with the size comparison task showed a reversed distance effect (poorer performance with the large compared with the small distance) in the incongruent condition, but only in the low and normal achievement groups. The authors interpreted the reversed task-irrelevant distance effect as automatic numerical processing (see also Tang et al., 2006). The lack of reversed distance effect in the high achievement group was due to shorter RTs in the long distance when trials were incongruent. This suggests that children in the high group were more efficient to solve conflict between the task-relevant and task-irrelevant dimensions in that particular condition, a result that can be interpreted as higher ability in inhibitory control to deal with conflict.

### The Current Study

In the present study we tested children from 6 to 11 years old and a group of undergraduates, a range of age that allowed us to trace the development of numerical processing and to investigate age-related changes in both intentional and automatic numerical processing (Rubinsten et al., 2002; Mussolin and Noël, 2007; Bugden and Ansari, 2011). The participants performed the two comparison tasks in one single experiment. In contrast to previous studies that investigated children’s mathematics performance by one or few tests, we selected five tests from the Spanish version of Woodcock Johnson III Tests of Achievement (Muñoz-Sandoval et al., 2005): Calculation, Fluency, Applied Problems, Concepts, and Series, the two latter as subtests of the test Quantitative Concepts. This allowed us to assess several levels of mathematical abilities that are related to relevant mathematical areas at school (see Materials and Methods).

On the basis of aforementioned related studies, we first expected our comparison tasks being sensitive to the developmental trajectory previously described. As we tested 6-year-old children at the beginning of first grade, automatic numerical processing, indexed by the numerical Stroop effect in the size comparison task, should not be observed at that age, although the effect is expected to manifest at the age of 7 years (Rubinsten et al., 2002). However, intentional numerical processing, as revealed by the distance effect in the numerical comparison task is expected to be present since the age of 6 years and on. Some studies have revealed that conflict scores that index executive control performance improves from 4 to around 7 years of age (Rueda et al., 2004, 2005) or even later (Casey et al., 2001), when it reaches adult-like levels. Thus, regarding inhibition of the task-irrelevant dimension, indexed by congruency effects in the numerical comparison task, we expected older participants to show greater efficiency in the control of task-irrelevant conflicting information. Younger children, however, should show more difficulties than older children to solve conflict from task-irrelevant incongruent physical size trials, due to immature attentional control at those ages.

Because to our knowledge, only few studies have explored the relationship between numerical processing and a rather large variety of mathematical abilities, no clear predictions can be raised respect to the relationship between the different numerical processing indexes and concrete mathematical areas. An inspection to the operations required by the different mathematical tests suggests that some abilities are based on exact basic arithmetic operations that may depend on recovery of number facts from long-term memory, like in the test Fluency (Andersson, 2008), or in the quick activation of the numerical magnitude of Arabic numerals, like in the test Calculation. Performance in intentional numerical processing may relate to the efficiency in such operations (Bugden and Ansari, 2011). In contrast, other mathematical abilities may make more demands on executive control processes such as those involved in the test Applied Problems, which requires both to hold information in memory and to integrate new information with the previously processed one (Swanson, 2011; Pina et al., 2014). Performance in both intentional and automatic numerical processing that requires high involvement of inhibitory processing (and therefore of executive control), may relate to the efficiency to solve word problems and/or numerical sequences that depend on similar cognitive capacity.

## Materials and Methods

### Participants

A sample of 109 typically developing primary school children was recruited from two suburban schools from the Region of Murcia (Spain), with socioeconomic level ranging from low to middle. Children with special educative needs, speech therapy, and intelligence scores below two standard deviations from the average, as well as bilingual speakers, were excluded from the study. The group of undergraduates were 33 (12 male; Mean age = 20.3; SD = 1.80) Psychology students at the Faculty of Psychology (University of Murcia, Spain). They performed only the two comparison tasks. Written informed consent from parents and oral consent from participants (only children), and informed consent from undergraduate students, were collected before the testing sessions. Demographic data from all participants are presented in **Table 1**.

**Table 1:** Descriptive data of participants.

Years	*n* (boys)	Age		
		*M*	SD	Range
6	18 (8)	76.3	3.9	72–83 (months)
7	12 (5)	91.3	4.4	85–95 (months)
8	27 (13)	101.6	3.5	96–107 (months)
9	23 (14)	112.3	3.5	108–119 (months)
10	19 (10)	125.3	3.1	120–130 (months)
11	10 (6)	136.4	3.4	132–142 (months)
Undergraduate	33 (12)	20.4	1.8	18–25 (years)

### General Procedure

The present study obtained the approval from the bioethics committee of the University of Murcia. Children were tested in two sessions. In the first session participants performed individually the intelligence and the mathematical tests featured in this study, and they were asked to reply in written or oral form to pencil and paper tests. In the second session they performed the two comparison tasks in groups of the same age, with a maximum of 12 children per group. We used the 12 computers located in the computer room available in the schools. One experimenter and six assistants stayed in the room during the testing session. The experimenter explained the instructions to the group of children through PowerPoint presentations. The assistants checked (one assistant each two children) that the participants had understood the task and completed it correctly. Undergraduate participants performed the two comparison tasks in a room with five computers located at the Faculty of Psychology. As with children, the instructions to perform the task were given by the experimenter through PowerPoint presentations. The tasks followed a counterbalanced sequence, which aimed to avoid systematic biases arising from the order of administration.

### Measures

#### Numerical Stroop

We used two computerized versions of the comparison tasks: the size comparison task and the numerical comparison task. Stimuli were presented on the 15^′′^ color monitors of the computers with Windows XP Professional at 1024 × 768 pixels resolution. E-Prime software was used to program the tasks (Schneider et al., 2002). The distance of the participant to the screen was approximately 60 cm.

All participants (children and undergraduates) completed two counterbalanced blocks of trials with the same stimuli: one for the size comparison task and the other for the numerical comparison task. In each trial two crosses separated by 8 cm appeared in the center of the screen for 1 s. Then, the two digits replaced the crosses and remained on until a response was emitted or until 5 s had elapsed. The right and left keys in a joystick were assigned to choose where the target digit (bigger in size or larger in numerical magnitude) was located. Each block started with 10 practice trials. Practice trials provided a smiley face for correct responses or a sad face for incorrect responses as feedback, and it was shown for 300 ms. Next, the experimental trials started but feedback was not included. A total of 96 experimental trials were administered in two blocks, one for each comparison task (48 trials per block). Each block contained 24 congruent trials (a pair of digits in which one was larger on both the relevant and irrelevant dimensions), and 24 incongruent trials (one digit was larger on one dimension but smaller on the other). The bigger digit of each pair was always twice (26 mm × 18 mm) the smaller digit (13 mm × 9 mm). On each block, each digit value and physical size appeared on both sides of the visual field an equal number of times. Digits from 1 to 9 were used. The following numerical distances were used: small distance (1–2, 1–3, 4–5, 4–6, 7–9, 8–9) and large distance (1–6, 1–7, 3–8, 2–8, 4–9, 3–9). Thus, for each block there were eight conditions (2 congruency conditions × 2 target positions × 2 distances) repeated six times.

#### Mathematical Abilities

We tested math abilities with the Spanish version of the Woodcock–Johnson III (WJ-III) Achievement (ACH) battery, which was -primarily validated for use with participants from 6 to 13 years in Spain (see Diamantopoulou et al., 2012). The battery consists of the following tests:

#### Calculation

This test measures the ability to perform simple mathematical computations including addition, subtraction, multiplication, and division that increase in difficulty as the test progresses. Poor performance in the test may be due to limited basic skills in mathematics, to a limited instruction level, or to inattention. The test consists of 45 problems of increasing complexity with no time limit. The test administration stops once the participant makes six consecutive mistakes. We used the summed scores of the test as a measure of calculation. Ascending scores indicate better performance. Internal consistency estimate obtained with our sample was α = 0.87.

#### Math Fluency

This test measures performance on mathematical operations and the fluency to operate with numbers through simple calculus operations such as addition, subtraction, and multiplication facts. A deficient performance in the test suggests limited basic skills in math or lack of automation. The test consists of 160 arithmetic problems, and participants are asked to solve as many as possible in a 3-min time limit. We used the summed scores as a measure of fluency. Ascending scores indicate better performance.

#### Quantitative Concepts

This test measures mathematical knowledge and quantitative reasoning. It consists of two subtests: Concepts and Series. In the subtest *Concepts*, participants are asked to count or identify numbers, shapes, and sequences, and to know mathematical formulas and terms. It consists of 34 items of increasing difficulty, which are read to the participant. Internal consistency of the subtest Concepts was α = 0.86. In the subtest *Series*, participants are asked to identify a pattern from a series of written numbers and provide the missing number in the series. It consists of 23 problems of ascending difficulty. Internal consistency of the subtest Series was α = 0.86. We used the summed scores of each subtest as a measure of *Quantitative Concepts*. A deficient performance on quantitative concepts suggests a limited vocabulary and/or insufficient conceptual development. Internal consistency of the test Quantitative Concepts was α = 0.92.

#### Applied Problems

This test measures quantitative reasoning, mathematical performance, and mathematical knowledge. Participants are asked to listen to the problem, identify the procedure to follow, and perform simple calculus operations. Children have to filter the appropriate information and exclude extraneous information. Poor performance on this test may be explained by limited mathematical skills, comprehension difficulties, or insufficient ability on mathematical reasoning. The test consists of 62 problems of ascending difficulty presented orally and visually to the participant, and the test administration stops once the participant makes six consecutive errors. We used the summed scores as a measure of applied problems. Internal consistency of the test Applied Problems was α = 0.90.

#### Mathematical Composite Measures

Compounds scores can be also computed by combining performance in some of the above tests. The two main mathematical composite measures are: (1) *Math Calculation Skill*, which refers to basics mathematical skills (it includes Calculation and Fluency), and (2) *Math Reasoning*, which refers to knowledge and math reasoning, and provides a global measure of problem solving, analysis, reasoning, and vocabulary (it includes Applied Problems and Quantitative Concepts).

The raw scores from the different tests were transformed into *W* scores (Woodcock and Dahl, 1971; Woodcock, 1978), which are based on Rasch measurement model (Rasch, 1960; Wright and Stone, 1979). Ascending scores indicate better performance.

#### General Cognitive Ability (IQ)

We assessed intelligence with the Spanish version of the Kaufman Brief Intelligence Test (Kaufman and Kaufman, 1990). The test consists of two main subscales: vocabulary and matrices. Ascending scores indicate higher intelligence. Internal consistency estimate obtained with our sample was α = 0.80.

### Statistical Analyses

Correct RTs below 200 ms or 2.5 SD above the mean for each participant and condition were discarded from the statistical analyses (0.27% of the trials). By subtracting incongruent and congruent conditions in the size comparison task, and small and large distance in the numerical comparison task we computed the numerical Stroop effect and the numerical distance effect, respectively. We assessed the effect of age on our dependent variables through analyses of variance (ANOVA) for both RTs and errors. Analyses were separated for each comparison task as each effect was assumed to tap a different kind of numerical processing.

Pearson correlation coefficients were also computed to assess the relationships between both intentional and automatic numerical processing markers and the scores in the intelligence and mathematical tests.

## Results

Data from the two comparison tasks are presented in **Table 2**. We analyzed both error percentage and mean RTs for correct responses.

**Table 2:** Percentage of errors and mean reaction time (RTs) as a function of age for the experimental conditions of the two comparison tasks.

	Numerical comparison task				Size comparison task			
	Congruent small distance	Congruent large distance	Incongruent small distance	Incongruent large distance	Congruent small distance	Congruent large distance	Incongruent small distance	Incongruent large distance
Age	% errors	% errors	% errors	% errors	% errors	% errors	% errors	% errors
6	7.87	4.63	18.98	11.57	5.09	3.70	5.56	5.09
7	7.64	2.08	19.44	21.53	5.56	8.33	11.11	9.03
8	6.79	2.16	18.83	13.58	2.78	3.70	5.86	4.32
9	7.97	2.54	19.93	8.70	2.90	1.81	4.71	7.61
10	6.58	2.19	19.30	9.65	4.39	1.75	5.26	7.46
11	4.17	2.50	17.50	10.83	0	4.17	5.00	10.00
Under graduate	2.78	0.25	7.83	3.79	0.51	0.25	2.27	2.27

	**Congruent/small distance**	**Congruent/large distance**	**Incongruent/small distance**	**Incongruent/large distance**	**Congruent/small distance**	**Congruent/large distance**	**Incongruent/small distance**	**Incongruent/large distance**
**Age**	***M* (SD)**	***M* (SD)**	***M* (SD)**	***M* (SD)**	***M* (SD)**	***M* (SD)**	***M* (SD)**	***M* (SD)**

6	1684 (493)	1493 (437)	1701 (374)	1544 (443)	995 (366)	925 (360)	914 (253)	972 (337)
7	1120 (185)	972 (110)	1238 (256)	1165 (190)	624 (105)	633 (105)	778 (212)	687 (121)
8	1067 (289)	1002 (316)	1226 (452)	1123 (304)	662 (205)	664 (235)	676 (204)	723 (272)
9	994 (409)	839 (267)	1048 (343)	955 (294)	527 (84)	542 (118)	582 (114)	555 (102)
10	719 (196)	721 (225)	897 (200)	772 (182)	498 (106)	513 (133)	534 (141)	547 (150)
11	685 (131)	671 (105)	815 (130)	759 (158)	524 (137)	518 (142)	563 (163)	575 (142)
Under graduate	587 (113)	528 (110)	630 (119)	580 (111)	380 (63)	374 (51)	409 (79)	410 (74)

### Numerical Comparison Task

Statistical analyses were performed through three-way mixed ANOVAs, with size congruency (congruent, incongruent) and numerical distance (small, large) as within-participants factors, and age (6, 7, 8, 9, 10, 11, undergraduates) as the between-participants factor.

**FIGURE 1:**
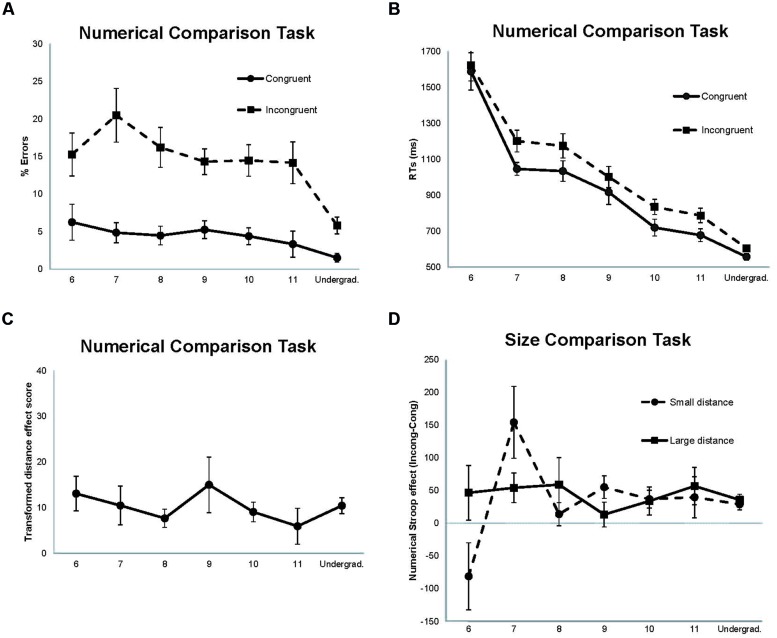
**Data from the comparison tasks. (A)** Percentage of errors for the size congruency conditions in the numerical comparison task. **(B)** Mean reaction times (RTs) for the size congruency conditions in the numerical comparison task. **(C)** Transformed scores for the distance effect in the numerical comparison task [(small distance RTs – large distance RTs)/large distance RTs]^∗^100. **(D)** Numerical Stroop effect as a function of numerical distance in the size comparison task (Incongruent RTs – Congruent RTs). Error bars (1 SE of the mean) are shown as vertical lines.

The error analysis showed significant main effects of size congruency [*F*(1,135) = 107.66, *p* < 0.00001; $\eta_{p}^{2}$ = 0.44], numerical distance [*F*(1,135) = 57.90, *p* < 0.00001; $\eta_{p}^{2}$ = 0.30], and age [*F*(6,135) = 4.88, *p* < 0.00001; $\eta_{p}^{2}$ = 0.18]. Larger percentage of errors was found in both incongruent and small distance conditions compared with congruent and large distance conditions. That is, the standard size congruency and numerical distance effects were observed. Also, undergraduates committed fewer errors than children, and children of different ages did not show significant differences in errors. However, the advantage of the undergraduates over the children was observed only in the incongruent condition, a result that was supported by the significant size congruency × age interaction [*F*(6,135) = 2.18, *p* = 0.049; $\eta_{p}^{2}$ = 0.09; **Figure 1A**]. None of the remaining interactions were statistically significant.

The RT analysis showed significant main effects of size congruency [*F*(1,135) = 68.21, *p* < 0.00001; $\eta_{p}^{2}$ = 0.34], numerical distance [*F*(1,135) = 44.88, *p* < 0.00001; $\eta_{p}^{2}$ = 0.25], and age [*F*(6,135) = 37.64, *p* < 0.00001; $\eta_{p}^{2}$ = 0.63]. RTs were shorter in the congruent condition compared with the incongruent condition (the size congruency effect), and in the large distance compared with the small distance (the standard numerical distance effect). RTs also decreased with age. The significant size congruency × age interaction [*F*(6,135) = 2.41, *p* = 0.030; $\eta_{p}^{2}$ = 0.10] revealed that size congruency effects were observed in all groups of age except in the youngest one (6 years; see **Figure 1B**).

Despite the distance effect ranged between 174 ms showed by 6-year-old children to 35 ms showed by 11-year-old children, the distance × age interaction was not significant [*F*(6,135) = 1.79, *p* = 0.106; $\eta_{p}^{2}$ = 0.07]. To check the possibility that variability in the RTs could have masked any difference in the distance effect as a function of age, we computed the individuals’ distance effect as a percentage of their large distance RT by using the following formula (see also Holloway and Ansari, 2009): (small distance RT – large distance RT)/large distance RT × 100. The results are shown in **Figure 1C**. Participants did not differ significantly in the distance effect with transformed data either (*F* < 1).

### Size Comparison Task

Statistical analyses were performed through three-way mixed ANOVAs, with numerical Stroop (congruent, incongruent) and numerical distance (small, large) as within-participants factors, and age (6, 7, 8, 9, 10, 11, undergraduates) as the between-participants factor.

The error analysis showed significant main effects of numerical Stroop [*F*(1,135) = 25.12, *p* < 0.00001; $\eta_{p}^{2}$ = 0.16], and age [*F*(6,135) = 4.76, *p* < 0.00001; $\eta_{p}^{2}$ = 0.17]. Participants were more accurate in the congruent condition than in the incongruent condition. Children of 7 years-old were less accurate than the rest of ages, and undergraduates were more accurate than the whole group of children (*p*s < 0.05).

The RT analysis showed significant main effects of numerical Stroop [*F*(1,135) = 23.28, *p* < 0.00001; $\eta_{p}^{2}$ = 0.15], and age [*F*(6,135) = 25.70, *p* < 0.00001; $\eta_{p}^{2}$ = 0.53]. Incongruent trials produced longer RTs than congruent trials, and the 6-year-old children and the undergraduates showed the longest and the shortest RTs, respectively. The numerical Stroop × age interaction was significant [*F*(6,135) = 2.28, *p* = 0.040; $\eta_{p}^{2}$ = 0.09], but this interaction was modulated by the significant three-way numerical Stroop × numerical distance × age interaction [*F*(6,135) = 2.85, *p* = 0.012; $\eta_{p}^{2}$ = 0.11]. The second order interaction was mainly due to a large reversed numerical Stroop effect in 6-year-old children (longer RTs in congruent than incongruent trials), and a large numerical Stroop effect in 7 year-old children, being both effects only observed with the small distance (*p*s < 0.05). However, the different age groups did not differ in the numerical Stroop effect with the large distance (*p* > 0.05; see **Figure 1D**). No other effects were found statistically significant.

### Mathematical Tests and Intelligence

Data are presented in **Table 3**. We performed one-way ANOVAs for each mathematical test, with age (6, 7, 8, 9, 10, and 11 years) as the between-participants factor. The results showed significant main effects of age on calculation [*F*(5,103) = 45.24, *p* < 0.00001; $\eta_{p}^{2}$ = 0.69], fluency [*F*(5,103) = 25.22, *p* < 0.00001; $\eta_{p}^{2}$ = 0.55], applied problems [*F*(5,103) = 26.96, *p* < 0.00001; $\eta_{p}^{2}$ = 0.57], quantitative concepts [*F*(5,103) = 28.06, *p* < 0.00001; $\eta_{p}^{2}$ = 0.58], math calculation skills [*F*(5,103) = 31.72, *p* < 0.00001; $\eta_{p}^{2}$ = 0.61], math reasoning [*F*(5,103) = 28.55, *p* < 0.00001; $\eta_{p}^{2}$ = 0.58], and intelligence [*F*(5,103) = 4.58, *p* = 0.001; $\eta_{p}^{2}$ = 0.18]. All the mathematical scores increased with children’s age except intelligence that decreased. However, all children showed intelligence scores within the normal range (92–107) according to the inclusion criteria. Accordingly, we included age and intelligence as control variables in the correlation analyses.

**Table 3:** Mean total scores as a function of age in mathematical and intelligence measures.

	Calculation	Fluency	Applied problems	Quantitative concepts	Math calculation skills	Math reasoning	IQ composite
Age	*M* (SD)	*M* (SD)	*M* (SD)	*M* (SD)	*M* (SD)	*M* (SD)	*M* (SD)
6	7.11 (2.93)	13.22 (11.48)	23.28 (4.59)	19.61 (4.67)	464.72 (17.67)	454.39 (16.56)	107.78 (12.98)
7	11.33 (1.82)	35.00 (7.71)	29.50 (2.39)	26.83 (5.11)	483.4 (5.48)	479.58 (12.59)	107.25 (12.85)
8	14.18 (2.91)	38.59 (10.46)	32.00 (4.51)	29.96 (4.97)	489.96 (7.19)	488.93 (15.3)	103.11 (13.30)
9	16.30 (3.01)	49.17 (16.93)	33.96 (4.54)	32.00 (5.81)	496.57 (8.47)	496.04 (16.18)	92.78 (11.09)
10	17.42 (2.22)	61.42 (19.26)	35.89 (3.83)	34.47 (3.13)	501.16 (6.61)	503.37 (10.10)	96.05 (12.45)
11	19.80 (2.25)	64.40 (23.75)	40.00 (4.83)	38.00 (3.50)	503.10 (13.94)	511.60 (19.82)	96.20 (12.89)

### Second-Order Correlations

Intelligence failed to correlate with any of the effects in the comparison tasks (*p*s > 0.05). However, in line with our previous findings, intelligence correlated with the mathematical tests (Pina et al., 2014; correlation coefficients ranged from *r* = 0.27 to *r* = 0.53; all *p*s < 0.01). **Table 4** shows the results of the partial correlations between the variables of interest controlled by both age and intelligence. The strength of the associations between the mathematics scores was medium to high, with significant correlation coefficients ranging from *r* = 0.43 to *r* = 0.87 (all *p*s < 0.00001).

**Table 4:** Correlations among all scores controlled by age and intelligence.

		1	2	3	4	5	6	7	8	9	10
Mathematics tests	(1) Calculation	1									
	(2) Fluency	0.54^∗∗^	1								
	(3) Applied problems	0.52^∗∗^	0.43^∗∗^	1							
	(4) Quantitative concepts	0.55^∗∗^	0.45^∗∗^	0.62^∗∗^	1						
	(5) Math calculation skills	0.83^∗∗^	0.62^∗∗^	0.45^∗∗^	0.49^∗∗^	1					
	(6) Math reasoning	0.59^∗∗^	0.48^∗∗^	0.87^∗∗^	0.87^∗∗^	0.58^∗∗^	1				
Numerical comparison Task (RTs)	(7) Distance effect – congruent	-0.06	-0.13	-0.09	-0.05	-0.07	-0.11	1			
	(8) Distance effect – incongruent	-0.15	-0.05	-0.21^∗^	-0.23^∗^	-0.03	-0.21^∗^	0.08	1		
Size comparison task (RTs)	(9) Congruency effect – large distance	0.09	0.13	0.19	0.14	0.15	0.17	-0.24^∗^	-0.10	1	
	(10) Congruency effect – small distance	0.21^∗^	0.19^∗^	0.14	0.12	0.34^∗∗^	0.16	-0.20^∗^	-0.01	0.04	1

^∗^*p* ≤ 0.05, ^∗∗^*p* ≤ 0.01.

When we compared the two comparison tasks, automatic and intentional numerical processing were only related when the physical size of the digits was congruent to the numerical value; that is, the RTs related to the numerical Stroop effect were negatively associated with the RTs related to the distance effect. In addition, we observed a dissociation in the relationship between each index of numerical processing and performance in the different mathematical tests (see **Table 4**).

The distance effect in the numerical comparison task correlated negatively with applied problems (*r* = -0.21; *p* = 0.028), quantitative concepts (*r* = -0.23; *p* = 0.019) and the composite math reasoning (*r* = -0.21; *p* = 0.030). That is, smaller distance effects were associated with better performance on those tests. Importantly, correlations were significant only when the task-irrelevant dimension of the stimuli (the physical size) was incongruent.

The numerical Stroop effect in the size comparison task correlated positively with calculation (*r* = 0.21; *p* = 0.027), fluency (*r* = 0.19; *p* = 0.046) and the composite mathematical skills (*r* = 0.34; *p* < 0.0001). That is, larger numerical Stroop effect was associated with better performance on those tests. Importantly, correlations were significant only when the two digits in the pair had a small distance.

## Discussion

In the current study we investigated: (1) age-related changes in both intentional and automatic numerical processing, from 6-year-old children to undergraduates; and (2) the relationships between math achievement and both intentional and automatic numerical processing. In contrast to some previous studies we tested children of an ample range of ages and used several mathematical tests assumed to tap an ample range of mathematical abilities.

In one single experiment participants responded either to the numerical value of a pair of digits, and therefore the physical size was task-irrelevant, or to the physical size and therefore the numerical value was task-irrelevant. The numerical comparison task allowed us to compute the numerical distance effect as an index of intentional numerical processing, and the size comparison task the numerical Stroop effect as an index of automatic numerical processing. In addition, children performed the mathematical tests, which allowed us to assess the relationships between both intentional and automatic numerical processing and the different mathematical abilities in children from 1 to 6 grades of primary school. We expected that by increasing the range of ages and the number of mathematical tests, we were able to depict a more detailed picture of how different forms of numerical processing relate to specific rather than general mathematical skills.

### Developmental Aspects of Numerical Processing

Regarding intentional numerical processing in the numerical comparison task, our data revealed that the distance effect was observed as early as 6 year-old and did not vary with age. These results are in line with previous studies that have observed intentional numerical processing in studies that compared 5 years-old children and adult participants, with both behavioral (Sekuler and Mierkiewicz, 1977; Duncan and McFarland, 1980; Siegler and Robinson, 1982; Huntley-Fenner, 2001; Fayol and Seron, 2005) and electrophysiological measures (Temple and Posner, 1998). The numerical comparison task allowed us to assess also inhibitory control abilities to deal with the task-irrelevant dimension (the physical size). Previous studies have shown that children aged 6 years manifest size congruency effects in the numerical comparison task, which means that the irrelevant physical size of the digits has been processed to some degree (Girelli et al., 2000; Rubinsten et al., 2002). However, according to previous studies, the reported size congruency effect seems to be mainly due to facilitation from congruent trials rather than interference from incongruent trials, when both conditions are compared with a neutral condition (e.g., both digits have the same size; see Rubinsten et al., 2002). Therefore, size congruency effects that are facilitatory based reflect that children at that age were able to process automatically the size of the stimuli. Although our current design did not include neutral trials and then facilitation and interference effects cannot be dissociated, the size congruency effect showed by our 6-year-old children was likely to be due to facilitation from congruent rather than interference from incongruent physical size, in line with the aforementioned studies. Inhibition abilities is not evident until the age of 7 years when complex tasks, such as those of the present study, are used (see Best and Miller, 2010, for a review).

Regarding automatic numerical processing in the size comparison task, the numerical Stroop effect varied depending on whether the numerical distance between the pair of digits was small or large. When the numerical distance was large, all the participants exhibited equivalent sizes of numerical Stroop effect. However, when the numerical distance was small age played a role in both the size and the direction of the numerical Stroop effect. A likely explanation for the differential pattern of numerical Stroop effects is that digits in the large irrelevant distance could be classified in a rather crude manner (e.g., large/small; Tzelgov et al., 1992; Girelli et al., 2000; Cohen Kadosh, 2008), which was similar across groups. In contrast, the small irrelevant numerical distance required more refined mapping between Arabic numerical representations and their magnitudes, due to the greater overlap between the number values.

Children aged 6 showed a large and reversed numerical Stroop effect with the irrelevant small distance; that is, congruent task-irrelevant numerical values produced longer RTs than incongruent numerical values. An inspection to **Table 2** reveals that extremely long RT in the congruent/small condition may have brought about the large reversed numerical Stroop effect. However, by looking at individual data from that age group we noticed that the reversed effect was caused mainly by four (out of 18) children with extremely high negative effects, ranging from -246 to -519 ms. It suggests that such reversed effect might not be a genuine effect. Therefore, we argue that children aged 6 years, when tested at the beginning of first grade, exhibit poor abilities in the automatic processing of exact numerical values when they activate overlapping numerical representations, likely due to scarce experience with numbers.

In contrast to 6-year-old children, older children have more experience with numbers, automatic processing becomes more established, and therefore interference from task-irrelevant numerical values should increase up to the point of stabilization. From a developmental point of view the stabilization level is reached when interference does not change as a function of age or other age-related conditions, something that we observed here with participants aged 8 years and on (see **Figure 1D**). However, interference effects may increase even when automatic numerical processing has reached the stabilization level if the inhibitory control system is inefficient to deal with conflict stemming from a task-irrelevant competitor. Children aged 7 years showed extremely large numerical Stroop effects with the irrelevant small distance (see **Figure 1D**). This result suggests that at the age of 7 years the inhibitory control system is not mature enough to deal with strong task-irrelevant competitors as it happens with overlapping numerical representations in the size comparison task (e.g., numerical values with small distance). However, at that age the inhibitory control system might be efficient enough to deal with weaker task-irrelevant competitors as it happens with the physical size of the digits in the numerical comparison task. Differences in the strength of both numerical and physical size dimensions are further supported by comparing performance in both tasks (see also Rubinsten et al., 2002). Responding to the numerical value of the digits takes longer and is less accurate (954 ms; 8.71% errors) than responding to their physical size (597 ms; 4.09% errors; see **Table 2**).

All the above results highlight the relevance of taking inhibitory processing into account when investigating the development of numerical processing (see also Houdé, 2000; Soltész et al., 2011; Houdé and Borst, 2014).

### Correlations Between the Numerical Processing Indexes

The numerical Stroop effect in the size comparison task correlated negatively with the distance effect in the numerical comparison task, but only when the distance effect was computed with congruent trials, which did not include conflicting information. The smaller the numerical distance effect, which indicates an efficient numerical processing, the higher the numerical Stroop effect in the size comparison task. This result suggests a link between intentional and automatic processing of numerical information, which fits well with theories of skill acquisition (Logan, 1988; Tzelgov et al., 2000).

It also agrees with the idea of a shared representational system between different types of numerical processing. Recent neuroimaging studies suggest that the posterior parietal cortex seems to be such shared system, which is activated by different magnitude representations such as time, size, quantity, and space (Fias et al., 2003; Kaufmann et al., 2005; Tang et al., 2006; Cohen Kadosh et al., 2007, 2008b; see Walsh, 2003 for review). Concretely, it is the right intra-parietal sulcus the brain area that is commonly activated by both automatic and intentional numerical processing (Cohen Kadosh et al., 2012).

### Numerical Processing as Predictor of Mathematical Performance

According to previous studies, inhibitory control may influence how numerical processing relates to different mathematical measures (Soltész et al., 2011; Gilmore et al., 2013; for a review see Friso-van den Bos et al., 2013). Inhibition may act by suppressing strategies not needed for the task in process. Thus, inhibition would suppress addition on a multiplication task, or irrelevant information on the process of solving a math problem. Our correlation analysis showed that both intentional and automatic processing predicted different mathematical abilities, but only when inhibitory control was also involved in the tasks.

#### Intentional Numerical Processing and Mathematical Performance

Regarding intentional numerical processing, a bulk of studies reported a negative correlation between the numerical distance effect and mathematical performance (De Smedt et al., 2009; Holloway and Ansari, 2009; Heine et al., 2010; Bugden and Ansari, 2011; Sasanguie et al., 2012; Vanbinst et al., 2012; Sasanguie et al., 2013), although only few studies included conflict from a task-irrelevant dimension in the experimental task (e.g., Heine et al., 2010). In the present study we found that some mathematical abilities were specifically related with intentional numerical processing. Our results showed that better intentional numerical processing predicts better performance in the mathematical abilities that involve mathematical reasoning, that is, applied word problems and quantitative concepts. These mathematical abilities have been shown to rely greatly on executive control functions (Pina et al., 2014).

In contrast to previous studies (e.g., Holloway and Ansari, 2009; Bugden and Ansari, 2011), we did not find any correlation between intentional numerical processing and rather basic mathematical operations (i.e., fluency). Differences in the involvement of inhibitory control in the numerical comparison task might explain the discrepancies. For instance, Bugden and Ansari (2011) did not manipulate the physical size of the digits in the numerical comparison task. Therefore, inhibitory control was not needed to deal with task-irrelevant information. In contrast, in the present study the inhibitory system was constantly required to be active to deal with conflict from a task-irrelevant dimension (i.e., the physical size). Thus, our numerical comparison task required the involvement of attentional control mechanisms that might have masked a relationship between pure intentional numerical processing and simpler mathematical abilities.

#### Automatic Numerical Processing and Mathematical Performance

In contrast to intentional numerical processing, automatic numerical processing related to operations that mainly rely on memory retrieval (e.g., fluency) and basic mathematical operations that require a quick activation of numerical magnitude represented by Arabic numerals (e.g., calculation). The fact that such relationships are found only when the numerical distance is small, suggests that such mathematical abilities may require automatic processing of exact numerical values and a better ability to deal with overlapping numerical representations.

## Conclusion

Our ample range of age and the study of different components of mathematics allowed us to reveal dissociable relationships between intentional and automatic numerical processing and specific rather than general mathematical abilities. The double dissociation observed here supports previous views of intentional processing as a marker of algorithmic processing, and automatic processing as a marker of memory retrieval and quick activation of semantic referents. These results extend that view to the field of numerical cognition and cognitive development. Also, the associations between the comparison task effects and mathematical scores occurred only when inhibitory processes were also involved, albeit the correlations were rather small. It suggests that inhibition skills may play an important role in predicting mathematical achievement beyond that predicted by how numerical information is processed (see Gilmore et al., 2013 for more direct evidence). These results highlight the relevance of inhibitory control in children’s mathematical achievement.

## Conflict of Interest Statement

The authors declare that the research was conducted in the absence of any commercial or financial relationships that could be construed as a potential conflict of interest.
